# Supplementary material for: Identification of Freezing-Responsive microRNAs and Their Targets in Chinese Jujube by Small RNA and Degradome Sequencing
Source: Int J Mol Sci. 2026 Sep 4;27(17):7912. doi: 10.3390/ijms27177912 (PMC13566890; doi:10.3390/ijms27177912)
Supplement: Supplementary file 1 [file ijms-27-07912-s001.zip › Supplementary Material S2/novel_miR_159.pdf]

```
Provisional ID            : novel_miR_159
Score total               :      0.3
Score for star read(s)    :     -1.3
Score for read counts     :       0
Score for mfe             :       0
Score for randfold        :      1.6
Score for cons. seed      :
Total read count          :     2579
Mature read count         :     2545
Loop read count           :       0
Star read count           :      34
```

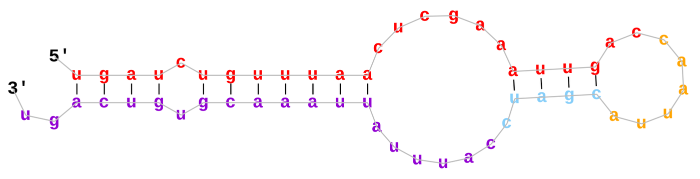


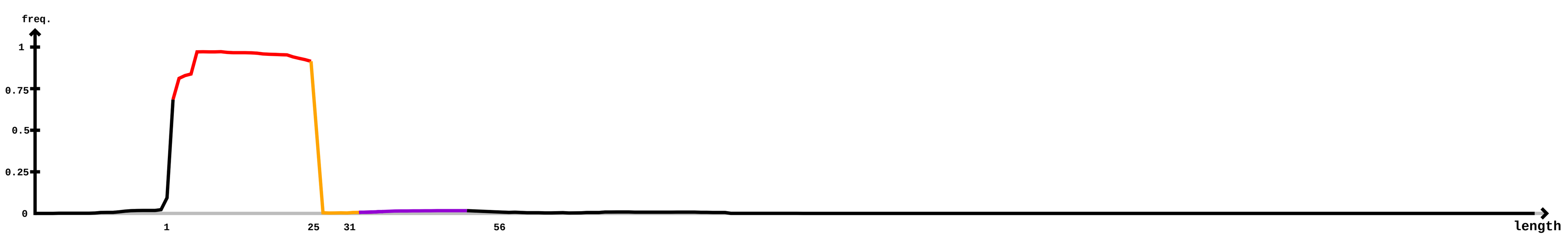


**Mature** **Star**

```
5'- uuaaucguguuauuuucggauugaucuguuuaacucgaaauugaccaauuacgauccauuuauuaaacgugucaguuucaacccaacacgauuauauaccuauuacaacccauuuaaauuuaauuuuaaauuaauauuuaucauuaauauaauauuuaauaacuaaaaaauauuauaaauuaaaaauuuuauaaaaauaauuuucuauuauuaauuuuuuaaaacaaaaaauacauuuuuaauaaaaca -3' obs
    uuaaucguguuauuuucggauugaucuguuuaacucgaaauugaccaauuacgauccauuuauuaaacgugucaguuucaacccaacacgauuauauaccuauuacaacccauuuaaauuuaauuuuaaauuaauauuuaucauuaauauaauauuuaauaacuaaaaaauauuauaaauuaaaaauuuuauaaaaauaauuuucuauuauuaauuuuuuaaaacaaaaaauacauuuuuaauaaaaca      exp
    .(((((((((......((((((((.(((((((......((((........))))......))))))).))))))))......)))))))))...............(((((((......)))))))...............((((((((((.........)))))))))).((((((((........((((((.....)))))).(((((((......)))))))..))))))))......  reads  mm  sample
    ..aaucguguuauuuuUggau....................................................................................................................................................................................................................................  1  1  LT-1
    ..aaucguguuauuuucggauugaucA..............................................................................................................................................................................................................................  2  1  JCK-2
    ........guuauuuucggauugaucuguu...........................................................................................................................................................................................................................  1  0  LT-1
    ........guuauuuucggauugaCcuguu...........................................................................................................................................................................................................................  2  1  LT-2
    .........uuauuuucggauugaCcug.............................................................................................................................................................................................................................  1  1  LT-1
    .........uuauuuucggauugaCcuguu...........................................................................................................................................................................................................................  1  1  LT-1
    .........uuauuuucggauugaucuguu...........................................................................................................................................................................................................................  4  0  LT-2
    .........uuauuuucggauugaCcuguu...........................................................................................................................................................................................................................  2  1  JCK-3
    .........uuauuuucggauugaCcuguu...........................................................................................................................................................................................................................  1  1  JCK-1
    ..........uauuuucggauugaucuguuu..........................................................................................................................................................................................................................  1  0  LT-1
    ............Guuucggauugaucuguuu..........................................................................................................................................................................................................................  4  1  LT-2
    ............uuuucggauugaucuguuuaac.......................................................................................................................................................................................................................  1  0  JCK-2
    ............Guuucggauugaucuguuuaacu......................................................................................................................................................................................................................  2  1  LT-3
    ............uuuucggauugaucuguuuaacuc.....................................................................................................................................................................................................................  1  0  LT-1
    ............uuuucggauugaucuguuuaacAc.....................................................................................................................................................................................................................  1  1  JCK-1
    .............uuucggauugaucuguuuaacu......................................................................................................................................................................................................................  1  0  JCK-3
    .............uuucggauugaucuguuuaacuc.....................................................................................................................................................................................................................  7  0  LCK-3
    .............uuucggauugaucuguuuaacuc.....................................................................................................................................................................................................................  1  0  LT-3
    .............uuucggauugaucuguuuaacucg....................................................................................................................................................................................................................  2  0  LT-1
    ..............uucggauugaucuguuuaacu......................................................................................................................................................................................................................  2  0  LT-3
    ..............uucggauugaucuguuuaacuc.....................................................................................................................................................................................................................  1  0  LT-3
    ..............uucggauugaucuguuuaacucA....................................................................................................................................................................................................................  1  1  LT-3
    ..............uucggauugaucuguuuaacucA....................................................................................................................................................................................................................  1  1  JCK-3
    ..............uucggauugaucuguuuaacucA....................................................................................................................................................................................................................  1  1  JCK-2
    ..............uucggauugaucuguuuaacucgC...................................................................................................................................................................................................................  1  1  JCK-1
    ...............ucggauugaucuguuuaac.......................................................................................................................................................................................................................  1  0  JCK-2
    ...............ucggauugaucuguuuaacuc.....................................................................................................................................................................................................................  1  0  LT-1
    ...............ucggauugaucuguuuaacucgaC..................................................................................................................................................................................................................  1  1  JCK-2
    ................cggauugaucuguuuaacu......................................................................................................................................................................................................................  1  0  LT-3
    ...................auugaucCguuuaacucga...................................................................................................................................................................................................................  1  1  JCK-2
    ...................auugaucCguuuaacucgaa..................................................................................................................................................................................................................  1  1  LT-3
    ...................auugaucuguuuaacucgaaa.................................................................................................................................................................................................................  1  0  JCK-2
    ...................auugaucuguuuaacucgaaau................................................................................................................................................................................................................  1  0  JCK-3
```

Mature Star

```
uuaaucguguuauuuucggauugaucuguuuaacucgaaauugaccaauuacgauccauuuauuaaacgugucaguuucaacccaacacgauuauauaccuauuacaacccauuuaaauuuaauuuuaaauuaauauuuaucauuaauauaauauuuaauaacuaaaaaauauuauaaauuaaaaauuuuauaaaaauaauuuucuauuauuaauuuuuuaaaaacaaaaaauacauuuuuaauaaaaaca
...................auugaucuguuuaacucgaaauC.................................................................................................................................................................................................   1     1     JCK-2
...................auugaucuguuuaacucgaaauC.................................................................................................................................................................................................   5     1     JCK-3
...................auugaucCguuuaacucgaaauug................................................................................................................................................................................................   2     1     LT-3
...................auugaucCguuuaacucgaaauug................................................................................................................................................................................................   1     1     LT-1
....................uugaucuguuuaacucga.....................................................................................................................................................................................................   1     0     LT-3
....................uugaucuguuuaacucgaaa...................................................................................................................................................................................................   2     0     JCK-1
....................uugaucuAuuuaacucgaaau..................................................................................................................................................................................................   3     1     JCK-3
....................uugaucuguuuaacucgaaau..................................................................................................................................................................................................   1     0     JCK-3
....................uugaucuAuuuaacucgaaau..................................................................................................................................................................................................   7     1     JCK-2
....................uugaucuguuuaacucgaaau..................................................................................................................................................................................................   1     0     LT-3
....................uugaucuguuuaacucgaaau..................................................................................................................................................................................................   2     0     JCK-2
....................uugaucuAuuuaacucgaaau..................................................................................................................................................................................................   3     1     LT-2
....................uugaucuAuuuaacucgaaau..................................................................................................................................................................................................   2     1     LCK-1
....................uugaucuguuuaacucgaaau..................................................................................................................................................................................................   2     0     LT-1
....................uugaucCguuuaacucgaaau..................................................................................................................................................................................................   3     1     LT-2
....................uugaucuAuuuaacucgaaau..................................................................................................................................................................................................   1     1     LT-1
....................uugaucuAuuuaacucgaaau..................................................................................................................................................................................................   2     1     JCK-1
....................uugaucCguuuaacucgaaau..................................................................................................................................................................................................   1     1     LCK-3
....................uugaucCguuuaacucgaaauu.................................................................................................................................................................................................   1     1     LT-2
....................uugaucCguuuaacucgaaauu.................................................................................................................................................................................................   2     1     JCK-3
....................uugaucCguuuaacucgaaauu.................................................................................................................................................................................................   2     1     JCK-1
....................uugaucuguuuaacucgaaauug................................................................................................................................................................................................   1     0     JCK-1
....................uugaucuguuuaacucgaaauug................................................................................................................................................................................................   1     0     JCK-2
....................uugaucuguuuaacucgaaauug................................................................................................................................................................................................   1     0     LT-3
....................uugaucuguuuaacucgaaauug................................................................................................................................................................................................   6     0     LCK-3
....................uugaucuguuuaacucgaaauugC...............................................................................................................................................................................................   1     1     JCK-2
....................uugaucCguuuaacucgaaauuga...............................................................................................................................................................................................   1     1     LT-3
....................uugaucuguuuaacucgaaauugac..............................................................................................................................................................................................   5     0     LT-1
....................uugaucuguuuaacucgaaauugac..............................................................................................................................................................................................   47    0     JCK-2
....................uugaucuguuuaacucgaaauuAac..............................................................................................................................................................................................   1     1     LT-3
....................uugaucuguuuaacucgaaauugac..............................................................................................................................................................................................   1     0     LT-3
....................uugaucuguuuaacucgaaauuAac..............................................................................................................................................................................................   3     1     LT-1
....................uugaucuguuuaacucgaaauugac..............................................................................................................................................................................................   1     0     LT-2
....................uugaucuguuuaacucgaaauugac..............................................................................................................................................................................................   43    0     JCK-1
....................uugaucuguuuaacucgaCauugac..............................................................................................................................................................................................   1     1     JCK-3
....................uugaucuguuuaacucgaaauugac..............................................................................................................................................................................................   41    0     JCK-3
.....................ugaucuguuuaacucgaC....................................................................................................................................................................................................   1     1     LT-1
.....................ugaucuguuuaacucgaa....................................................................................................................................................................................................   1     0     LT-3
.....................ugaucuguuuaacucgaaauuga...............................................................................................................................................................................................   1     0     LT-3
.....................ugaucuguuuaCcucgaaauugac..............................................................................................................................................................................................   1     1     JT-1
.....................ugaucuguuuaacucgaaauugac..............................................................................................................................................................................................   4     0     JT-2
.....................uNaucuguuuaacucgaaauugac..............................................................................................................................................................................................   1     1     JCK-3
.....................ugaucuguuuaacucgaaauuNac..............................................................................................................................................................................................   1     1     JCK-1
.....................ugaucuguuuaacucgaaauugac..............................................................................................................................................................................................   61    0     LT-1
.....................uUaucuguuuaacucgaaauugac..............................................................................................................................................................................................   1     1     JCK-3
.....................ugaucuguuuaacucgaaauuAac..............................................................................................................................................................................................   2     1     LT-3
.....................ugaucuguuuaacucgaaauugac..............................................................................................................................................................................................   431   0     JCK-3
.....................ugaucuguuuaacucCaaauugac..............................................................................................................................................................................................   5     1     JCK-2
.....................Cgaucuguuuaacucgaaauugac..............................................................................................................................................................................................   1     1     JCK-1
.....................ugaucCguuuaacucgaaauugac..............................................................................................................................................................................................   1     1     LT-3
.....................ugaucuguuuaacucgaaauugac..............................................................................................................................................................................................   20    0     LT-2
.....................ugaucuguuuaacucgaaauugac..............................................................................................................................................................................................   5     0     JT-3
.....................ugaucuguuuaacucgaaauugac..............................................................................................................................................................................................   50    0     LCK-3
.....................ugaucuguuuaacucgaaauugac..............................................................................................................................................................................................   18    0     JT-1
.....................ugaucuguuuaacucCaaauugac..............................................................................................................................................................................................   1     1     LT-1
.....................ugaucuguuuaacucCaaauugac..............................................................................................................................................................................................   6     1     JCK-3
.....................ugaucuguuuaacucgaaauugac..............................................................................................................................................................................................   323   0     JCK-1
.....................Cgaucuguuuaacucgaaauugac..............................................................................................................................................................................................   3     1     JCK-3
.....................ugCucuguuuaacucgaaauugac..............................................................................................................................................................................................   1     1     JCK-3
.....................ugaucuguuuaNcucgaaauugac..............................................................................................................................................................................................   1     1     JCK-1
.....................ugaucuguuuaacucgaGauugac..............................................................................................................................................................................................   1     1     JCK-1
.....................ugaucuguuuaacucCaaauugac..............................................................................................................................................................................................   1     1     LT-2
.....................ugaucuguuuaacucgaaauuAac..............................................................................................................................................................................................   4     1     LT-1
.....................Cgaucuguuuaacucgaaauugac..............................................................................................................................................................................................   3     1     JCK-2
.....................ugaucuguuuaacucgaaauugac..............................................................................................................................................................................................   566   0     JCK-2
.....................ugaucuguuuaacucgaaauugac..............................................................................................................................................................................................   34    0     LT-3
.....................ugaucCguuuaacucgaaauugac..............................................................................................................................................................................................   5     1     JCK-3
.....................ugaucuguuuCacucgaaauugac..............................................................................................................................................................................................   1     1     JCK-3
.....................ugaucCguuuaacucgaaauugac..............................................................................................................................................................................................   2     1     JCK-2
.....................ugaucuguuuaacucgaaauuAac..............................................................................................................................................................................................   6     1     LT-2
```

```
                     Mature           Star
uuaaucguguuauuuucggauugaucuguuuaacucgaaauugaccaauuacgauccauuuauuaaacgugucaguuucaacccaacacgauuauauaccuauuacaacccauuuaaauuuaauuuuaaauuaauauuuaucauuaauauaauauuuaauaacuaaaaaaauauuauaaauuaaaaauuuuauaaaaauaauuuucuauuauuaauuuuuuaaaaacaaaaaauacauuuuuaauaaaaca
.....................ugaucuguuuaacucgaaauugaA....    1    1    JCK-3
.....................ugauAuguuuaacucgaaauugac....    1    1    JCK-3
.....................ugaucuguuuaacucCaaauugac....    5    1    JCK-1
.....................ugaucuguuuaacucgaaauugacA....   1    1    JCK-1
.....................ugaucuguuuaacucgaaauugacU....   1    1    LT-1
.....................ugaucuguuuaacucgaaauugacc....   1    0    JCK-1
.....................ugaucuguuuaacucgaaauugacc....   1    0    JCK-2
......................gaucuguuuaacucgaaau....        2    0    LT-2
......................gaucuguuuCacucgaaau....        1    1    LT-2
......................gaucuguuuaacucgaaauuga....     1    0    LT-3
......................gaucuguuuaacucgaaauugac....    12   0    LCK-3
......................gaucuguuuaacucgaaauugac....    10   0    JT-1
......................gCucuguuuaacucgaaauugac....    1    1    JT-1
......................gaucuguuuaacucgaaauugaU....    1    1    JCK-2
......................gaucuguuuaacucgaaauugac....    33   0    JCK-2
......................gCucuguuuaacucgaaauugac....    1    1    JCK-1
......................gaucCguuuaacucgaaauugac....    1    1    JCK-1
......................gCucuguuuaacucgaaauugac....    1    1    JCK-2
......................gaucuguuuaacucgaaauugac....    35   0    JCK-1
......................gaucuguuuaacucgaCauugac....    1    1    JCK-3
......................gaucuguuuaacucgaaauugac....    34   0    JCK-3
......................gaucCguuuaacucgaaauugac....    1    1    JCK-3
......................gaucuguuuaacucgaaauugac....    2    0    LT-3
......................gaucuguuuaacucgaaauuAac....    1    1    LT-1
......................Aaucuguuuaacucgaaauugacc....   1    1    JCK-1
......................gNucuguuuaacucgaaauugacc....   1    1    JCK-1
......................gaucuguuuaacucgaaauugacc....   1    0    LT-2
......................gCucuguuuaacucgaaauugacc....   1    1    JCK-2
......................gaucuguuuaacucgaaauugacc....   57   0    JCK-3
......................gaucuguuuaacucgaaauugacc....   46   0    JCK-1
......................gaucuguuuaacucgaCauugacc....   1    1    JCK-2
......................gaucuguuuaacucgaaauugacc....   66   0    JCK-2
......................gaucuguuuaacucgaaauuAacc....   1    1    LT-3
......................gaucuguuuaacucgaaauugacc....   7    0    LCK-3
......................gaucuguuuaacucgaaauugaUc....   7    1    LCK-3
......................gaucuguuuaacucgaaauugacc....   8    0    LT-1
......................gaucuguuuaacucgaaauugaGc....   1    1    JCK-2
......................gaucuguuuaacucgaaauugaGc....   2    1    JCK-1
.......................aucugCuuaacucgaaauu....       5    1    LCK-3
.......................aucuguuuaacucgaaauu....       4    0    LCK-3
.......................Uucuguuuaacucgaaauugac....    5    1    LCK-3
.......................aucuguuuaacucgaaauugac....    14   0    LCK-3
.......................aucuguuuaacucgaaauugacc....   1    0    LT-1
.......................aucuguuuaacucgaaauugaUc....   2    1    LCK-3
.......................aucuguuuaacucgaaauugacc....   4    0    JCK-3
.......................aNcuguuuaacucgaaauugacc....   1    1    JCK-1
.......................aucuguuuaacucgaaauuAacc....   1    1    LT-1
.......................aucuguuuaacucgaaauugacc....   2    0    JCK-1
.......................aucuguuuaacucgaaauugacc....   2    0    JCK-2
.......................aucuguuuaacucgaaauugacca....  2    0    JT-3
.......................aucuguuuaacucgaaauugacca....  2    0    JT-2
........................ucuguuuaacucgaaauu....       1    0    LT-3
........................ucuguuuaacucgaaauu....       2    0    LT-1
........................ucuguuuaacucgaaauuga....     7    0    LT-2
........................ucuguuuaacucgaaauuga....     1    0    LT-3
........................ucuguuuaacucgaaauugac....    1    0    JCK-3
........................ucuguuuaacucgaaauugac....    1    0    JCK-2
........................ucuguuuaacucgaaauugac....    5    0    LT-3
........................ucuguuuaacucgaaauugacc....   1    0    JCK-1
........................ucuguuuaacucgaaauugacc....   1    0    JCK-3
........................ucuguuuaacucgaaauugacc....   3    0    JCK-2
........................ucuguuuaacucgaaauugacc....   1    0    LT-1
........................ucuguuuaacucgaaauugacc....   1    0    LT-3
.........................cuguuuaacucgaaauug....      1    0    JCK-1
.........................cuguuuaacucgaaauuC....      5    1    LT-2
.........................cuguuuaacucgaaGuug....      3    1    LT-2
.........................cuguuuaacucgaaauuga....     10   0    LCK-3
.........................cuguuuaacucgaaauuga....     2    0    LT-3
.........................cuguuuaacucgaaauuga....     3    0    LT-1
.........................cuguuuaacucgaaauugac....    52   0    LCK-1
```

**Mature** **Star**

| | | | |
|---|---|---|---|
| uuaaucguguuauuuucggauugaucuguuuaacucgaaauugaccaauuacgauccauuuauuaaacgugucaguuucaacccaacacgauuauauaccuauuacaacccauuuaaauuuaauuuuaaauuaauauuuaucauuaauauaauauuuaauaacuaaaaaauauuauaaauuaaaaauuuuauaaaaauaauuuucuauuauuaauuuuuuaaaaacaaaaaauacauuuuuaauaaaaca | | | |
| .........................cuguuuCacucgaaauugac | 1 | 1 | JCK-3 |
| .........................cuguuuaacucgaaauugac | 5 | 0 | JCK-1 |
| .........................cuguuuaacucgaaauugac | 48 | 0 | LCK-2 |
| .........................cuguuuaacucgaaauugCc | 1 | 1 | JCK-1 |
| .........................cuguuuaacucgaaNuugac | 1 | 1 | LCK-1 |
| .........................cuguuuaacucgaaauugac | 32 | 0 | LCK-3 |
| .........................cuguuuaacucgaaauugac | 46 | 0 | LT-2 |
| .........................cuguuuaacucgaaauugac | 7 | 0 | JCK-3 |
| .........................cuguuuaacucgaaauugac | 15 | 0 | JCK-2 |
| .........................Uuguuuaacucgaaauugac | 1 | 1 | LT-3 |
| .........................cuguuuaacucgaaauugac | 9 | 0 | LT-1 |
| .........................Auguuuaacucgaaauugac | 1 | 1 | LCK-2 |
| .........................cuguuuaacucgaaauugac | 12 | 0 | LT-3 |
| .........................cuguuuCacucgaaauugac | 1 | 1 | LCK-2 |
| .........................cuguuuaacucgaaauugacU | 4 | 1 | LCK-3 |
| .........................cuguuuaacucgaaauugacc | 11 | 0 | LT-3 |
| .........................cuguuuaacucgaaauugacc | 5 | 0 | LCK-3 |
| .........................cuguuuaacucgaaauugacU | 6 | 1 | LT-3 |
| .........................cuguuuaacucgaaauugacU | 7 | 1 | LT-2 |
| .........................cuguuuaacucgaaauugacc | 6 | 0 | JCK-2 |
| .........................cuguuuaacucgaaauugacU | 1 | 1 | JCK-2 |
| .........................cuguuuaacucgaaauugacc | 4 | 0 | JT-3 |
| .........................cuguuuaacucgaaauugacU | 1 | 1 | JCK-3 |
| .........................cuguuuaacucgaaauugacc | 2 | 0 | JCK-3 |
| .........................cuguuuaacucgaaauugacU | 2 | 1 | LT-1 |
| .........................cuguuuaacucgaaauugacc | 3 | 0 | JCK-1 |
| .........................Uuguuuaacucgaaauugacc | 2 | 1 | LT-3 |
| .........................Uuguuuaacucgaaauugacc | 5 | 1 | LT-2 |
| .........................Auguuuaacucgaaauugacc | 1 | 1 | JCK-1 |
| .........................cuguuuaacucgaaauugacc | 16 | 0 | LT-1 |
| .........................cuguuuaacucgaaauugacc | 22 | 0 | LT-2 |
| ..........................uguuuaacucgaaauugacU | 2 | 1 | LCK-3 |
| ............................Cuuaacucgaaauugaccaa | 1 | 1 | LT-1 |
| .............................uuaacucgaaauugaccaauu | 2 | 0 | JCK-2 |
| .............................uuaacucgaaauugaccaauu | 1 | 0 | JCK-1 |
| .............................................caauuacgauccauuuau | 2 | 0 | LT-2 |
| ................................................uuacgauccauuuauuaaacAug | 1 | 1 | JCK-3 |
| ................................................uuaUgauccauuuauuaaacgugu | 1 | 1 | LT-1 |
| .................................................uacgauccauuuauuaaacguguc | 2 | 0 | LT-1 |
| ..................................................aUgauccauuuauuaaacgugu | 1 | 1 | LT-3 |
| ...................................................cgauccauuuauuaaacgug | 2 | 0 | LT-1 |
| ...................................................cgauccauuuauuaaacgugu | 3 | 0 | LT-2 |
| ...................................................cgauccauuuauuaaacgugu | 1 | 0 | LT-1 |
| ...................................................cgauccauuuauuaaacguguU | 1 | 1 | LT-1 |
| ....................................................gGuccauuuauuaaacgugucagu | 2 | 1 | JCK-1 |
| .....................................................auUcauuuauuaaacgugucaguu | 1 | 1 | JCK-1 |
| .....................................................auUcauuuauuaaacgugucaguu | 3 | 1 | JCK-3 |
| .......................................................ccauuuauuaaacgugucag | 4 | 0 | LCK-3 |
| .......................................................ccauuuauuaaacgugucagu | 1 | 0 | LT-3 |
| ........................................................cauuuauuaaUcgugucagu | 5 | 1 | JCK-2 |
| .........................................................auuuauuaaacgugucaguuuc | 1 | 0 | JCK-3 |
| .........................................................auuuauuaaacgugucaguuuc | 2 | 0 | JCK-2 |
| .........................................................auuuauuaaacgugucaguuuc | 1 | 0 | JCK-1 |
| ..........................................................uuuauuaaacgugucaguuuca | 4 | 0 | LT-2 |
| ...........................................................uuauuaaacgugucagCuucaacc | 1 | 1 | LT-3 |
| .............................................................auuaaacgugucaguuucaacU | 2 | 1 | LT-2 |
| ...............................................................Aaaacgugucaguuucaacccaac | 3 | 1 | JCK-2 |
| .................................................................aacgugucaguuucaacccaac | 2 | 0 | LT-1 |
| ..............................................................................caacccaacacgauuauauU | 3 | 1 | LCK-3 |
| .....................................................................................acacgauuauauaGcuauuacaac | 1 | 1 | JCK-1 |
| .....................................................................................acacgauuauauaGcuauuacaac | 1 | 1 | JCK-2 |
| ......................................................................................cacgauGauauaccuauuacaac | 2 | 1 | JCK-2 |
| ........................................................................................cgauuauauaccuauuacaaccc | 1 | 0 | LT-1 |
| .........................................................................................gauuauauaccuauuacaaccc | 1 | 0 | LT-3 |
| ..........................................................................................auuauauaccuauuacaacccauu | 2 | 0 | JCK-3 |
| ..........................................................................................auuauauaccuauuacaacccauu | 3 | 0 | JCK-2 |
| .............................................................................................auauaccuauuacaacccauu | 4 | 0 | LCK-3 |
| .............................................................................................auauaccuauuacaacccauu | 1 | 0 | LT-1 |
| .............................................................................................auauaccuauuacaacccauu | 3 | 0 | LT-2 |
| ...............................................................................................auaUcuauuacaacccauu | 1 | 1 | LT-1 |

```
                     Mature                       Star
uuaaucguguuauuuucggauugaucuguuuaacucgaaauugaccaauuacgauccauuuauuaaacgugucaguuucaacccaacacgauuauauaccuauuacaacccauuuaaauuuaauuuuaaauuaauauuuaucauuaauauaauauuuaauaacuaaaaaauauuauaaauuaaaaauuuuauaaaaauaauuuucuauuauuaauuuuuuaaaaacaaaaaauacauuuuuaauaaaaca
.........................................................................................................caacccauuuaaauuuaauuu............................................................................................................................    1    0    JCK-2
```
